# Supplementary material for: Tead1a Initiates Transcriptional Priming Through the TEAD1a/YAP‐Notch1‐Spi1/Cebpα Axis to Promote Neutrophil Fate
Source: Adv Sci (Weinh). 2025 Aug 28;12(41):e05441. doi: 10.1002/advs.202505441 (PMC12591127; doi:10.1002/advs.202505441)
Supplement: Supplementary file 4 — Supplementary Dataset [file ADVS-12-e05441-s004.zip › 3-4.zip!/3-4/02-4.cluster/cluster11/Enrich/ego_MF.pdf]

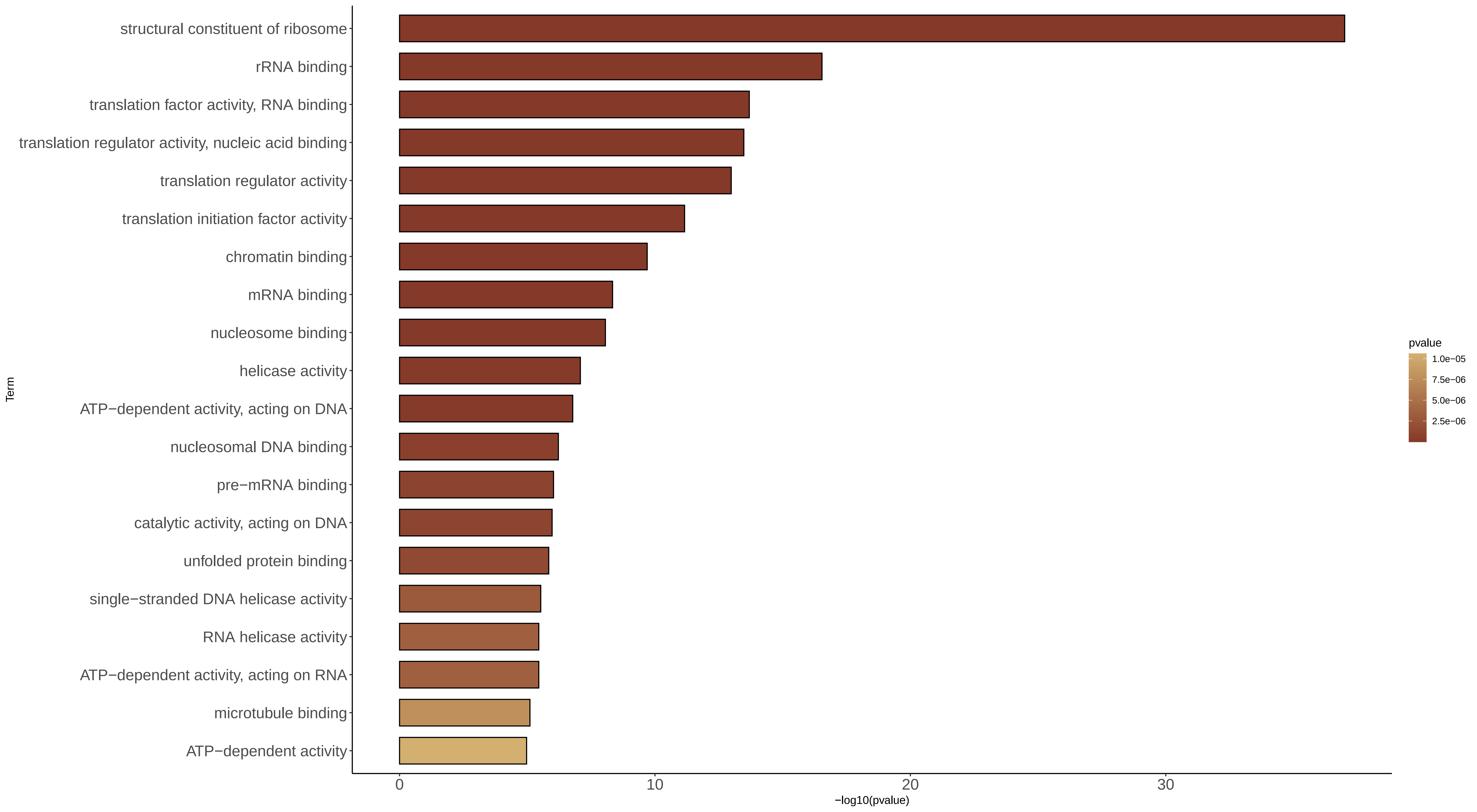

structural constituent of ribosome
rRNA binding
translation factor activity, RNA binding
translation regulator activity, nucleic acid binding
translation regulator activity
translation initiation factor activity
chromatin binding
mRNA binding
nucleosome binding
helicase activity
ATP−dependent activity, acting on DNA
nucleosomal DNA binding
pre−mRNA binding
catalytic activity, acting on DNA
unfolded protein binding
single−stranded DNA helicase activity
RNA helicase activity
ATP−dependent activity, acting on RNA
microtubule binding
ATP−dependent activity
Term
0
10
20
30
−log10(pvalue)
pvalue
1.0e−05
7.5e−06
5.0e−06
2.5e−06
